# Clinicopathologic and molecular spectrum of *RNASEH1*-related mitochondrial disease

**DOI:** 10.1212/NXG.0000000000000149

**Published:** 2017-05-02

**Authors:** Enrico Bugiardini, Olivia V. Poole, Andreea Manole, Alan M. Pittman, Alejandro Horga, Iain Hargreaves, Cathy E. Woodward, Mary G. Sweeney, Janice L. Holton, Jan-Willem Taanman, Gordon T. Plant, Joanna Poulton, Massimo Zeviani, Daniele Ghezzi, John Taylor, Conrad Smith, Carl Fratter, Meena A. Kanikannan, Arumugam Paramasivam, Kumarasamy Thangaraj, Antonella Spinazzola, Ian J. Holt, Henry Houlden, Michael G. Hanna, Robert D.S. Pitceathly

**Affiliations:** From the MRC Centre for Neuromuscular Diseases (E.B., O.V.P., A.M., A.H., J.L.H., H.H., M.G.H., R.D.S.P.), UCL Institute of Neurology and National Hospital for Neurology and Neurosurgery; Department of Molecular Neuroscience (A.M., A.M.P., J.L.H., H.H., M.G.H.), Division of Neuropathology (J.L.H.), Department of Clinical Neuroscience (J.-W.T., A.S., I.J.H.), UCL Institute of Neurology; Neurometabolic Unit (I.H.), Neurogenetics Unit (C.E.W., M.G.S.), Department of Neuro-ophthalmology (G.T.P.), National Hospital for Neurology and Neurosurgery, London; Nuffield Department of Obstetrics and Gynaecology (J.P.), University of Oxford; MRC-Mitochondrial Biology Unit (M.Z.), Cambridge, UK; Unit of Molecular Neurogenetics (D.G.), Fondazione IRCCS Istituto Neurologico “Carlo Besta,” Milan, Italy; Oxford Medical Genetics Laboratories (J.T., C.S., C.F.), Oxford University Hospitals NHS Foundation Trust, Churchill Hospital, UK; Department of Neurology (M.A.K.), Nizam's Institute of Medical Sciences; CSIR-Centre for Cellular and Molecular Biology (A.P., K.T.), Hyderabad, Telangana, India; MRC Mill Hill Laboratory (I.J.H.), London, UK; Biodonostia Research Institute (I.J.H.), San Sebastián, Spain; and Department of Basic and Clinical Neuroscience (R.D.S.P.), Institute of Psychiatry, Psychology and Neuroscience, King's College London, UK.

## Abstract

**Objective:**

Pathologic ribonuclease H1 (RNase H1) causes aberrant mitochondrial DNA (mtDNA) segregation and is associated with multiple mtDNA deletions. We aimed to determine the prevalence of RNase H1 gene (*RNASEH1*) mutations among patients with mitochondrial disease and establish clinically meaningful genotype-phenotype correlations.

**Methods:**

*RNASEH1* was analyzed in patients with (1) multiple deletions/depletion of muscle mtDNA and (2) mendelian progressive external ophthalmoplegia (PEO) with neuropathologic evidence of mitochondrial dysfunction, but no detectable multiple deletions/depletion of muscle mtDNA. Clinicopathologic and molecular evaluation of the newly identified and previously reported patients harboring *RNASEH1* mutations was subsequently undertaken.

**Results:**

Pathogenic c.424G>A p.Val142Ile *RNASEH1* mutations were detected in 3 pedigrees among the 74 probands screened. Given that all 3 families had Indian ancestry, *RNASEH1* genetic analysis was undertaken in 50 additional Indian probands with variable clinical presentations associated with multiple mtDNA deletions, but no further *RNASEH1* mutations were confirmed. *RNASEH1*-related mitochondrial disease was characterized by PEO (100%), cerebellar ataxia (57%), and dysphagia (50%). The ataxia neuropathy spectrum phenotype was observed in 1 patient. Although the c.424G>A p.Val142Ile mutation underpins all reported *RNASEH1*-related mitochondrial disease, haplotype analysis suggested an independent origin, rather than a founder event, for the variant in our families.

**Conclusions:**

In our cohort, *RNASEH1* mutations represent the fourth most common cause of adult mendelian PEO associated with multiple mtDNA deletions, following mutations in *POLG*, *RRM2B*, and *TWNK*. *RNASEH1* genetic analysis should also be considered in all patients with *POLG*-negative ataxia neuropathy spectrum. The pathophysiologic mechanisms by which the c.424G>A p.Val142Ile mutation impairs human RNase H1 warrant further investigation.

Mitochondrial diseases are commonly inherited disorders caused by mutations in nuclear and mitochondrial DNA (mtDNA). Dysfunction in a subset of nuclear genes involved with mtDNA maintenance results in the secondary accumulation of multiple deletions and/or depletion of muscle mtDNA.^1^ The clinical spectrum of this group of disorders is broad, ranging from severe infantile hepatocerebral syndromes to benign, late-onset progressive external ophthalmoplegia (PEO). Mutations in the mtDNA maintenance gene *RNASEH1*, encoding ribonuclease H1 (RNase H1), have recently been linked to adult mitochondrial disease presenting with CNS and neuromuscular involvement.^2^ RNase H1 degrades RNA hybridized to DNA,^3^ and knockout mice suffer embryonic lethality owing to mtDNA depletion,^4^ attributable to persistent mtDNA RNA/DNA hybrids.^5^ However, humans with *RNASEH1* mutations develop a relatively mild clinical syndrome, comprising adult-onset PEO associated with multiple mtDNA deletions secondary to the impaired physical segregation of mtDNA molecules.^6^ The prevalence and clinical spectrum of *RNASEH1*-related mitochondrial disease is currently unknown.

We screened 74 unrelated probands with genetically undetermined mitochondrial disease for *RNASEH1* mutations. The previously reported pathogenic missense transition c.424G>A p.Val142Ile was detected in 3 families with Indian ancestry: homozygous mutations were confirmed in 2 pedigrees and compound heterozygous mutations in combination with a novel c.442T>C p.Cys148Arg variant in a third. Detailed clinicopathologic and molecular profiling of these newly identified families harboring *RNASEH1* mutations, and evaluation of all previously reported cases, was subsequently undertaken to determine the phenotypic spectrum of *RNASEH1*-mediated mitochondrial disease and establish clinically meaningful genotype-phenotype correlations. Finally, the ancestral origins of the “common” *RNASEH1* c.424G>A p.Val142Ile mutation were investigated.

## METHODS

### Participants.

Patients referred to the London and Oxford NHS England nationally commissioned service for mitochondrial diseases were recruited to the study according to the following criteria: (1) confirmed multiple deletions/depletion of muscle mtDNA and (2) mendelian PEO with pathologic evidence of mitochondrial dysfunction, including ragged red fibers (RRFs) and/or cytochrome *c* oxidase (COX)-negative muscle fibers, without detectable multiple deletions or depletion of muscle mtDNA. *RNASEH1* genetic analysis was also undertaken in a cohort of Indian patients with evidence of multiple mtDNA deletions detectable in muscle.

### Genetic studies.

Whole-exome and Sanger sequencing was used to analyze the 8 coding exons and intron-exon boundaries of *RNASEH1* (primer sequences available on request).

### Clinicopathologic and molecular evaluation of patients.

Patients harboring *RNASEH1* mutations were assessed clinically by the authors. Medical records and muscle tissue histopathologic and electron microscopy findings were reviewed. Real-time quantitative PCR of DNA extracted from muscle was undertaken in all patients harboring *RNASEH1* mutations and multiple mtDNA deletions to exclude coexisting mtDNA depletion. Previously published *RNASEH1* variants (pathogenic and of unknown significance) were evaluated^2,7^ and clinicopathologic and genotypic data extracted and combined with the London-Oxford cohort to accurately define the clinical genetic spectrum of all known *RNASEH1*-mediated mitochondrial disease.

### Principal component analysis, identity by descent, and haplotyping.

Principal component analysis (PCA), using GCTA software (version 1.26.0), was performed relative to the HapMap project populations (CEU, JPT, and YRI) to study the geographical origin of 2 Indian families where exome-sequencing data were available (families A and B). Identity by descent (IBD) analysis was utilized in plink software (v1.90 b1g) to explore the possibility of a common lineage for the pedigrees. Haplotyping was adopted to investigate the possibility of a common origin for the c.424G>A p.Val142Ile mutation. Six single nucleotide variants extracted from available exome data were used to construct haplotypes around the mutation site, and Sanger sequencing of the variants in affected individuals from the London-Oxford cohort and in 2 previously reported families was undertaken.^6^ Haplotypes were phased around homozygous variant sites only, given parental samples were unavailable.

### Standard protocol approvals, registrations, and patient consents.

The study was approved and performed under the ethical guidelines issued by our institutions for clinical studies, with written informed consent obtained from all participants for genetic studies.

## RESULTS

### *RNASEH1* sequence data analysis.

**Figure 1:**
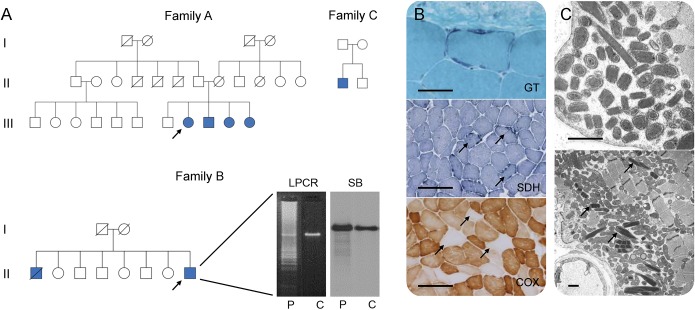
Pedigrees and histopathologic findings of patients harboring *RNASEH1* c.424G>A p.Val142Ile mutations (A) Pedigrees of families harboring *RNASEH1* mutations. Filled blue symbols represent affected individuals. Arrows indicate probands. Long range PCR (LPCR) and Southern blot (SB) demonstrate multiple deletions of muscle mitochondrial DNA (B-II.8) in patient (P) when compared with control muscle (C). (B) Muscle biopsy histology (A-III.8) demonstrating ragged red fibers (modified Gomori trichrome, GT), ragged blue fibers (succinate dehydrogenase, SDH), and several muscle fibers deficient in cytochrome *c* oxidase (COX), arrows. Scale bar represents 50 μm in GT and 200 μm in SDH and COX. (C) Ultrastructural examination (A-III.8) showing increased numbers of mitochondria, many of which are structurally abnormal, including the presence of paracrystalline inclusions, arrows. Scale bar represents 1 μm.

Seventy-four unrelated probands were recruited from the London and Oxford NHS England nationally commissioned service for mitochondrial diseases and categorized as follows: multiple deletions (n = 33) and depletion (n = 21) of muscle mtDNA and mendelian PEO with neuropathologic evidence of mitochondrial dysfunction, but no detectable multiple deletions/depletion of muscle mtDNA (n = 20). Homozygous *RNASEH1* c.424G>A p.Val142Ile mutations were identified in 2, apparently unrelated, nonconsanguineous families (family A: A-III.8, A-III.9, A-III.10, and A-III.11; and family B: B-II.1 and B-II.8), and a third singleton case (family C: C-II.1) was compound heterozygous with the novel missense mutation c.442T>C p.Cys148Arg (figure 1A). Parental segregation was confirmed. All 3 families had Indian ancestry. Affected individuals harbored multiple deletions of muscle mtDNA. Fifty additional unrelated Indian probands with multiple deletions of muscle mtDNA deletions were screened for *RNASEH1* mutations (see table e-1 at Neurology.org/ng, for clinicopathologic spectrum), but no known or novel *RNASEH1* variants were detected.

### Clinicopathologic and molecular features in *RNASEH1* mutations.

Mean age of symptom onset in the London-Oxford cohort was 29 years (range, 13–36 years). All patients presented with either ptosis or imbalance. The clinical phenotype was characterized by PEO, proximal muscle weakness, and cerebellar ataxia. One patient had a sensory ataxic neuropathy, with nerve conduction studies (NCS) and EMG indicative of a nonlength-dependent, predominantly sensory, axonal neuropathy. Brain MRI demonstrated moderate generalized parenchymal volume loss (n = 2). Histopathologic examination of muscle revealed RRFs and COX-negative fibers in all cases examined, while ultrastructural examination showed increased and abnormal mitochondria, many with paracrystalline inclusions (figure 1, B and C). There was no evidence of a coexisting reduction in muscle mtDNA copy number (B-II.8 and C-II.1).

Three families (6 affected individuals) with confirmed *RNASEH1* mutations have previously been reported.^2^ An additional case was identified from variants of unknown significance published in exome-sequencing studies.^7^ These data were combined with the London-Oxford cohort and confirmed that PEO was a universal feature in patients with *RNASEH1*-related mitochondrial disease and that a substantial proportion of patients (57%) exhibited cerebellar dysfunction. Additional clinical features (summarized in table 1) included dysphagia (50%), proximal muscle weakness (36%), peripheral neuropathy (36%), and pyramidal signs (14%). NCS and EMG were consistent with involvement of motor and/or sensory nerve fibers with demyelinating, axonal, or ganglionic features. RRFs and/or COX-deficient fibers and multiple mtDNA deletions were reported in all cases when muscle tissue was available.

**Table 1:**
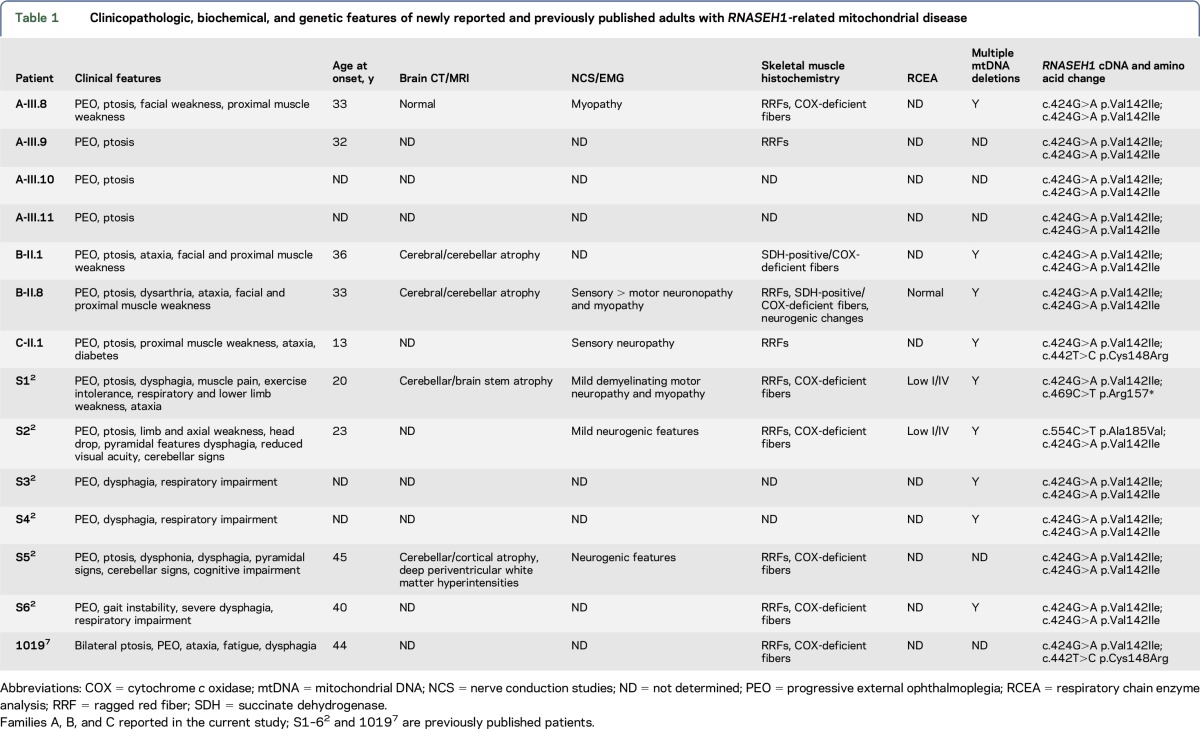
Clinicopathologic, biochemical, and genetic features of newly reported and previously published adults with *RNASEH1*-related mitochondrial disease

The c.424G>A p.Val142Ile mutation was detected in all 7 families (both newly reported and previously published). Three were homozygous and 4 were compound heterozygous with the following mutations: c.442T>C p.Cys148Arg (n = 2), c.469C>T p.Arg157*, and c.554C>T p.Ala185Val.

### PCA, IBD, and haplotyping.

**Figure 2:**
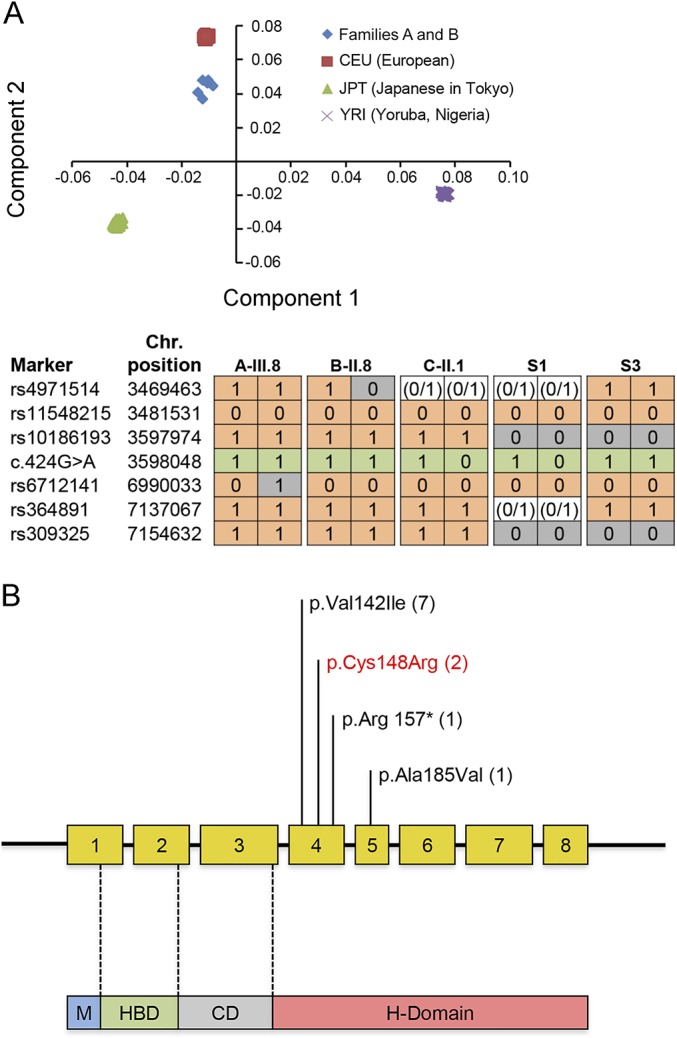
Principal component and haplotype analysis of families harboring c.424G>A p.Val142Ile variant and schematic illustrating the newly identified and previously reported *RNASEH1* mutations to date (A) Principal component analysis (top panel). X-axis represents component 1; Y-axis represents component 2. Families A and B cluster to the same ethnic group. Haplotype analysis of individuals harboring the *RNASEH1* mutation c.424G>A p.Val142Ile (bottom panel). “1” indicates the presence of the marker, “0” indicates the absence of the marker, and “0/1” is used when haplotype could not be phased. Haplotypes reported are for A-III.8, B-II.8, C-II.1, S-1, and S-3. Green = c.424G>A mutation; gray = markers differing from reference haplotype. Families A and B shared a haplotype of at least 3.57 Mb. However, 2 additional haplotypes were different, suggesting distant recombination events or that the haplotypes arose independently. (B) The *RNASEH1* gene has 8 exons encoding 4 protein domains as follows: mitochondrial targeting sequencing domain (M), hybrid-binding domain (HBD), connection domain (CD), and ribonuclease H domain (H-domain). The latter conducts all catalytic activity. Both herein newly identified and previously reported mutations are illustrated in the schematic. To date, all affected individuals harbor the “common” c.424G>A p.Val142Ile mutation either in homozygous or heterozygous states. Brackets = number of families with each mutation. Red = newly reported variant.

PCA revealed that families A and B clustered to the same ethnic group (figure 2A, top panel), while IBD analysis implied a distant relationship to approximately second cousins (mean PI_HAT 0.049 ± 0.013). Haplotyping of families A and B suggested one shared founder haplotype of 3.67 Mb which was also present in family C. However, given that the c.424G>A p.Val142Ile variant was heterozygous, it was not possible to decipher whether this represented a population-specific or an ancestral mutation (figure 2A, bottom panel). Analysis of the same haplotype marker set in 2 previously reported pedigrees with European ancestry (S1 and S3) revealed a different haplotype at single nucleotide polymorphism rs10186193, 74 base pairs away from the c.424G>A p.Val142Ile mutation. These data are consistent with an independent origin for the c.424G>A p.Val142Ile mutation in the patients analyzed.

## DISCUSSION

Based on data obtained from patients referred to the London and Oxford NHS England nationally commissioned service for mitochondrial diseases, *RNASEH1* mutations represent the fourth most common cause of mendelian PEO associated with multiple mtDNA deletions in adults (2.7%, 3/109), following mutations in *POLG* (24.7%, 27/109), *RRM2B* (16.5%, 18/109), and *TWNK* (also known as *C10orf2*, *PEO1*, or Twinkle, 16.5%, 18/109). Additional, but less frequent, causes of adult mendelian PEO associated with multiple mtDNA deletions in our cohort include *SLC25A4* (*ANT1*), *OPA1*, *MFN2*, *TYMP*, *TK2*, and *SPG7* (1/109, 0.9%), while *AFG3L2*, *DNA2*, *MGME1*, *POLG2*, *DGUOK*, and *MPV17* were not detected.

Two families, in whom affected individuals harbored multiple mtDNA deletions, were homozygous for the previously reported c.424G>A p.Val142Ile mutation, thus confirming its pathogenicity and the importance of RNase H1 in mtDNA maintenance. A third proband was compound heterozygous with a novel missense mutation c.442T>C p.Cys148Arg in a highly conserved region of the enzyme, resulting in a hydrophobic amino acid being replaced by a positively charged residue within the RNase H1 active site. This mutation was previously reported as a variant of unknown significance in a patient with PEO harboring the *POLG2* variant c.1105A>G p.Arg369Gly, which was favored as being causative.^7^ However, a minor allele frequency of 0.003 suggests that the *POLG2* variant c.1105A>G p.Arg369Gly is a benign polymorphism and unlikely to be deleterious.^8^ Furthermore, the similarity in clinical phenotypes and association with identical mutations in our cohort supports the pathogenic basis of *RNASEH1* c.442T>C p.Cys148Arg when a second mutant allele is present.

Clinically, *RNASEH1*-linked mitochondrial disease is relatively homogenous, comprising PEO, cerebellar ataxia, dysphagia, and proximal muscle weakness. This could reflect similar structural and functional consequences of the reported *RNASEH1* mutations. Indeed, all patients are homozygous or heterozygous for c.424G>A p.Val142Ile, and remaining variants all occur within in the same catalytic domain (figure 2B). Additional deleterious mutations associated with more severe, early-onset clinical phenotypes might exist. However, we did not identify any cases in our mtDNA depletion cohort. Furthermore, it is possible that very detrimental mutations are embryonic lethal as is seen in knockout mice which are completely deficient in RNase H1.^4^ Finally, sensory ataxic neuropathy, previously unreported with *RNASEH1* mutations, expands the recognized clinical phenotype to include ataxia neuropathy spectrum, most commonly associated with recessive *POLG* mutations.^9^

IBD analysis confirmed that families A and B were distantly related. However, their haplotype differed from 2 previously reported European pedigrees.^2^ These data suggest that the c.424G>A p.Val142Ile mutation has arisen independently in the lineages analyzed. European admixing of the London-Oxford Indian families harboring c.424G>A p.Val142Ile could account for the lack of *RNASEH1* mutations in the 50 additional Indian probands screened. Furthermore, the close proximity of all 3 families to the CEU (European) HapMap project populations (figure 2A) suggests that they belong to the Ancestral North Indian gene pool, which shares up to 70% genetic affinities with Europeans.^10^

Our data confirm that *RNASEH1* mutations are an important cause of mitochondrial disease resulting from the secondary accumulation of multiple mtDNA deletions and that the phenotypic spectrum in adults is relatively benign. Nevertheless, *RNASEH1* genetic analysis should be considered in all patients presenting with ataxia neuropathy spectrum once *POLG* mutations have been excluded. Finally, although the commonly occurring c.424G>A p.Val142Ile *RNASEH1* variant could indicate a mutation hotspot within the gene, it might instead reflect an inability of the mutant enzyme to bind with a partner protein, thereby allowing Val142Ile RNase H1 to attack its substrates indiscriminately.^6^ As such, loss-of-function mutations could confer different clinical phenotypes.

## Supplementary Material

Data Supplement

## GLOSSARY

COX: cytochrome c oxidase
IBD: identity by descent
mtDNA: mitochondrial DNA
NCS: nerve conduction studies
PCA: principal component analysis
PEO: progressive external ophthalmoplegia
RNase H1: ribonuclease H1
RRF: ragged red fiber
