# Supplementary material for: Clinicopathologic and molecular spectrum of RNASEH1-related mitochondrial disease
Source: Neurol Genet. 2017 May 2;3(3):e149. doi: 10.1212/NXG.0000000000000149 (PMC5413961; doi:10.1212/NXG.0000000000000149)
Supplement: Data Supplement [file supp_3_3_e149__index.html]

Data Supplement 

# Clinicopathologic and molecular spectrum of *RNASEH1*-related mitochondrial disease

## Data Supplement

**Files in this Data Supplement:**

- Table e-1
