# Supplementary material for: Clinicopathologic and molecular spectrum of RNASEH1-related mitochondrial disease
Source: Neurol Genet. 2017 May 2;3(3):e149. doi: 10.1212/NXG.0000000000000149 (PMC5413961; doi:10.1212/NXG.0000000000000149)
Supplement: Data Supplement [file supp_3.3.e149_Table_e-1.docx]

**Table e-1:** Clinicopathological spectrum of 50 unrelated Indian probands with multiple deletions of muscle mitochondrial DNA

| **Clinical phenotype** | **Probands** | **COX staining performed** | **COX negative fibers present** |
| --- | --- | --- | --- |
| PEO | 7 | 4 | 4 |
| PEO plus | 28 | 11 | 11 |
| Myopathy | 11 | 7 | 7 |
| Myopathy and parkinsonism | 2 | 2 | 2 |
| Myoclonus and cerebellar ataxia | 2 | 2 | 2 |

Abbreviations: COX = cytochrome *c* oxidase; PEO = progressive external ophthalmoplegia. COX negative fibers present in all muscle biopsies when COX staining performed.
